# Development and Characterization of Monoclonal Antibodies to Botulinum Neurotoxin Type E

**DOI:** 10.3390/toxins11070407

**Published:** 2019-07-13

**Authors:** Candace S. Bever, Miles Scotcher, Luisa W. Cheng, Robert M. Hnasko, Larry H. Stanker

**Affiliations:** 1 Foodborne Toxin Detection and Prevention Research Unit, Agricultural Research Service, United States Department of Agriculture, 800 Buchanan Street, Albany, CA 94710, USA; 2 Produce Safety and Microbiology Research Unit, Agricultural Research Service, United States Department of Agriculture, 800 Buchanan Street, Albany, CA 94710, USA

**Keywords:** monoclonal antibodies, botulinum neurotoxin serotype E, BoNT/E, ELISA, immunoassay

## Abstract

Botulism is a devastating disease caused by botulinum neurotoxins (BoNTs) secreted primarily by *Clostridium botulinum*. Mouse bioassays without co-inoculation with antibodies are the standard method for the detection of BoNTs, but are not capable of distinguishing between the different serotypes (A–G). Most foodborne intoxications are caused by serotypes BoNT/A and BoNT/B. BoNT/E outbreaks are most often observed in northern coastal regions and are associated with eating contaminated marine animals and other fishery products. Sandwich enzyme-linked immunosorbent assays (ELISAs) were developed for the detection of BoNT/E3. Monoclonal antibodies (mAbs) were generated against BoNT/E3 by immunizing with recombinant peptide fragments of the light and heavy chains of BoNT/E3. In all, 12 mAbs where characterized for binding to both the recombinant peptides and holotoxin, as well as their performance in Western blots and sandwich ELISAs. The most sensitive sandwich assay, using different mAbs for capture and detection, exhibited a limit of detection of 0.2 ng/ml in standard buffer matrix and 10 ng/mL in fish product matrices. By employing two different mAbs for capture and detection, a more standardized sandwich assay was constructed. Development of sensitive and selective mAbs to BoNT/E would help in the initial screening of potential food contamination, speeding diagnosis and reducing use of laboratory animals.

## 1. Introduction

Botulinum neurotoxins (BoNT) represent a family of toxins produced primarily by *Clostridium botulinum* and are the cause of botulism in both animals and humans. Botulism is a severe neurological disease that if untreated is often fatal. Seven toxin serotypes (BoNT/A–G) have been described, as well as potentially type H and a tentatively-named type X that are less well studied [1,2]. All BoNTs have a molecular weight of about 150 kDa and consist of two polypeptide chains, a light chain (LC) ~50 kDa and a heavy chain (HC) ~100 kDa linked via a single disulfide bridge. 

BoNT serotypes A and B, are responsible for the majority (90%) of confirmed human botulism cases in the U.S. followed by serotype E responsible for 10% (3 of 29) of the foodborne cases reported in 2016 [3]. Within the US and abroad, serotype E is the most prevalent toxin type identified in fish, fishery products, and aquatic environments, including the shoreline soil [4,5,6]. BoNT/E outbreaks have also been found in water birds in the Great Lakes, USA [7]. In Alaska, BoNT/E is the most frequent serotype, accounting for 82% (249 of 303) of the foodborne cases from 1950–2016 [8]. The propensity for exposure to BoNT/E in Alaska is likely due to the documented presence of *C. botulinum* type E spores within the environment, as well as cultural preferences for food preparation procedures for aquatic animals (i.e., butchering animals in coastal environments, as well as “fermenting” fish and sea mammals) [6,8,9,10,11]. Specifically, whales, seals, and Salmon (including Salmon eggs) are frequently involved.

All of the BoNT serotypes are immunologically distinct, and therefore antibody-based reagents can be used to distinguish each serotype. Mono- and polyclonal antibodies (mAbs and pAbs) possess unique specificities for their targets and allow for the identification of particular serotypes. Thus far, a wide variety of antibody reagents specific for BoNT/E have been generated, such as rabbit pAbs [12,13], horse pAbs [14], mouse mAbs [15,16,17,18], humanized single-chain variable fragments (scFvs) [19], and single-domain heavy chain-only antibodies (VHH) [20,21]. 

Antibody-based methods can be used for the detection of BoNTs in food and other biological samples. Highly selective and sensitive antibodies are employed in enzyme-linked immunosorbent assays (ELISAs), electrochemiluminescence immunoassays, lateral flow tests, and flow cytometry, as well as incorporated with other technologies, such as immuno-polymerase chain reaction, endopep-mass spectrometry combined with antibody capture, and fluorescence resonance energy transfer assays with immunocapture [22,23,24]. In most configurations, antibodies capable of binding the toxin in the solution phase (i.e., as a capture antibody) are the most suitable for sample analysis. Moreover, assays that utilize mAbs as both the capture and the detector antibodies are the most desirable because of the consistency and longevity in the supply of those reagents as opposed to using a pAb as either a capture or detector, since pAbs have a more limited supply. 

A key consideration is that not all immunoreagents are transferrable among different assay platforms. For example, some reagents can be used in sandwich assays, but do not bind immobilized toxin-complexes, and vice-versa [16,25]. Furthermore, some mAbs that do not react to the native toxin in a detection platform, possess neutralizing activity. Likewise, many of the mAbs used to develop detection tools to BoNT/E have also been used to analyze the structure of the isotype [26,27] and to elucidate toxin-neutralizing activity [16,19,28]. These situations are not uncommon and therefore a suite of antibodies with varying characteristics are needed in order to develop assays for a wide range of applications to advance our understanding of BoNT structure/function relationships. 

In this article, we show that we have expanded the repertoire of available and reproducible immunoreagents specific for BoNT/E. Our objective was to develop a sandwich ELISA for BoNT/E3, thus requiring the identification of two separate mAbs to work as a capture-detector pair. We applied our previously described strategy of using recombinant peptide fragments as immunogens [29,30], and constructed a series of peptide fragments for both the light chain (LC) and heavy chain (HC) regions of BoNT/E3. Screening procedures utilizing both direct binding and capture-capture methods were used to increase the possibility of isolating antibodies to the solution phase toxin. Binding of these newly generated mAbs to both BoNT/E and other BoNT serotypes was evaluated by Western blot and ELISA. Finally, we applied the sandwich ELISA to the detection of BoNT/E spiked in food matrices.

## 2. Results

### 2.1. Isolation and Characterization of Monoclonal Antibodies

**Figure 1:**
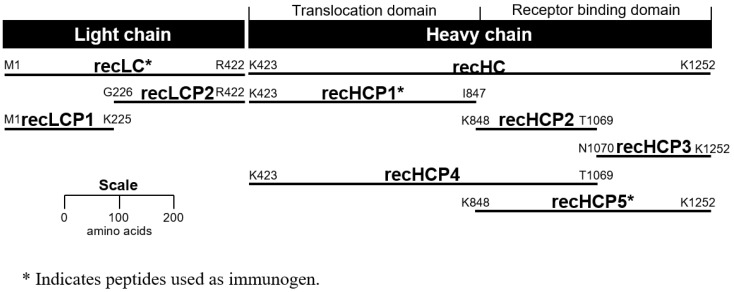
Peptide fragments of botulinum neurotoxins (BoNT)/E3. Glutathione S-transferase (GST) was fused on the N-terminus of each peptide.

Mice were immunized with the following recombinant glutathione S-transferase (GST) fusion peptides: recBoNT-LC-GST, recBoNT-HCP1-GST, and recBoNT-HCP5-GST (Figure 1). These correspond to the intact LC, the translocation domain, and the receptor binding domain, respectively. Two fusion screening assays were utilized to identify antibody producing hybridoma cells. For screening the LC-immunized mice, an ELISA in which purified BoNT/E complex was immobilized onto microtiter plates by adsorption was used. For screening the HC-immunized mice, a capture-capture ELISA was used. Goat anti-mouse immunoglobulin (Fc-specific) was absorbed onto microtiter plates, hybridoma supernatants were then applied, followed by the addition of BoNT/E3 toxin. Toxin captured from solution by putative mAbs secreted from the newly formed hybridoma cells was detected with a rabbit anti-BoNT/E pAb followed by a horseradish peroxidase (HRP)-conjugated anti-rabbit pAb.

A total of 12 stable mAb producing hybridoma cell lines were established following at least two cloning steps by limited dilution. They were designated as BoE 33-10, BoE 33-12, BoE 33-13-17-9, BoE 33-13-17-4, BoE 34-16, BoE 9-15, BoE 66-29, BoE 66-81, BoE 66-106, BoE 71-9, BoE 71-14, and BoE 71-95. The recombinant immunogen employed to produce each of these antibodies, the screening method used, the myeloma fusion partner, and the antibody heavy chain isotype (all possessed kappa light-chains) are summarized in Table 1.

### 2.2. Antibody Specificity

**Figure 2:**
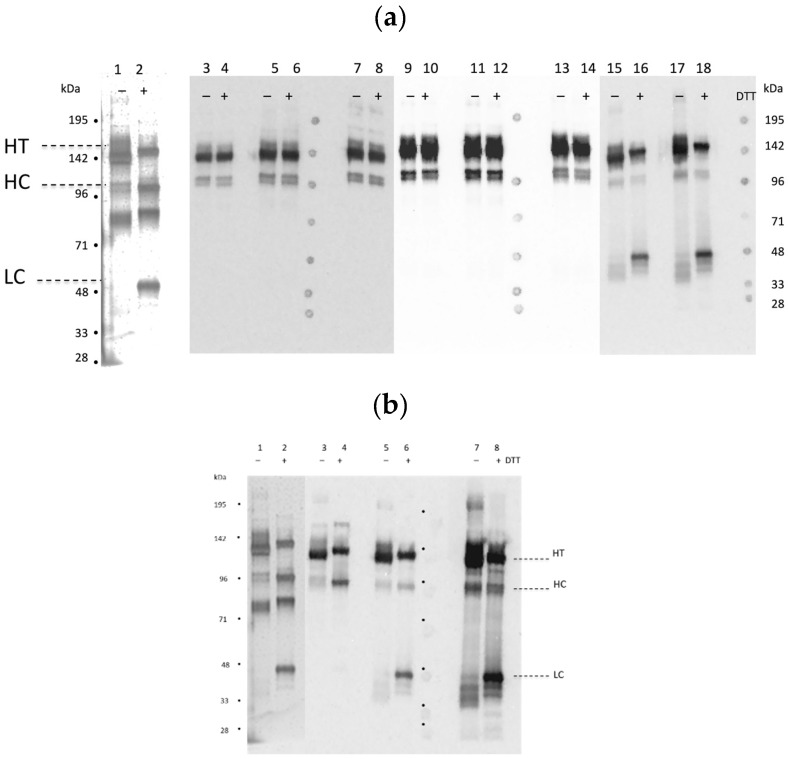
Western blots of BoNT/E3 nicked holotoxin with and without dithiothreitol (DTT). Bands (HT = holotoxin; HC = heavy chain; LC = light chain) were visualized by the following: (**a**) lanes 1–2, silver stain; lanes 3–4, BoE 71-9; lanes 5–6, BoE 71-14; lanes 7–8, BoE 71-95; lanes 9–10, BoE 66-29; lanes 11–12, BoE 66-81; lanes 13–14, BoE 66-106; lanes 15–16, BoE 33-13-17-4; lanes 17–18, BoE 34-16; (**b**) lanes 1–2, silver stain; lanes 3–4 BoE 9-15; lanes 5–6, BoE 33-10; 7–8 BoE 33-12. (BoE 33-17-9 not shown). The silver stained (lanes 1–2) band ~80 kDa is unknown/unidentified material not recognized by the antibodies.

Even though recombinant peptides were used to generate the antibodies, the binding specificity to neurotoxin HC vs. LC was confirmed by examining their binding profiles on reduced and non-reduced Western blots following electrophoresis of BoNT/E3 nicked holotoxin (Figure 2a,b). As expected, those mAbs isolated following immunization with recBoNT/E3-LC-GST peptide preferentially bound the toxin LC, with little binding to the HC bands (Figure 2b, lanes 15–18; and Figure 2b, lanes 5–8, respectively). Likewise, following sample reduction, antibodies produced using recombinant peptide fragments corresponding to regions of the toxin HC (HCP1 and HCP5) bound the HC with no binding to the LC (Figure 2a,b). All of the mAbs bound intact holotoxin in these Western blot experiments.

Antibody binding to BoNT serotypes A–G was evaluated using immobilized holotoxin in ELISA. These data are summarized in Table 1 and Figures S1 and S2. The mAbs generated to LC fragments fall into two distinct binding groups. The first group (comprising mAbs BoE 33-10, BoE 33-12, BoE 33-13-17-9, and BoE 34-16) bound multiple BoNT serotypes with the strongest binding to serotypes E,F, moderate binding to serotypes B,C,D, and G, and virtually no binding to serotype A (except for BoE 33-12). In sharp contrast, the second group of toxin LC binders is comprised of just one mAb (BoE 33-13-17-4) which demonstrated strong binding only to BoNT/E3 holotoxin (weak binding to serotype B was consistently observed), but no binding to serotypes A,C,D, and F (Figure S1C). All of the BoNT HC specific mAbs, were specific for BoNT/E3 with the exception of mAb BoE 66-106 that showed weak but consistent reactivity to BoNT serotype B as well as strong binding to serotype E3 (Figure S2).

### 2.3. Binding Epitopes

In addition to the peptides generated for use as immunogens, peptides were generated to half of the LC (recLCP1 and recLCP2), half of the receptor binding domain (recHCP2 and recHCP3), as well as one that spans the translocation domain and N-terminal half of the receptor binding domain (recHCP4) (Figure 1). Screening of the mAbs against all recHC and recLC peptides permitted more detailed analysis of binding activity. Screening was examined by ELISA and the results are summarized in Table 2 and Figure S3. As expected, those antibodies that were generated using the recLc immunogen bound only the recLC, specifically the recLCP1 peptide (corresponding to the N-terminal half of the LC). Although these mAbs all bound recLCP1, our data indicate that the mAbs generated to LC fragments either bind multiple toxin serotypes (mAbs BoE 33-10, BoE 33-12, and BoE 34-16) or bind only serotype E (mAb BoE 33-13-17-4).

**Figure 3:**
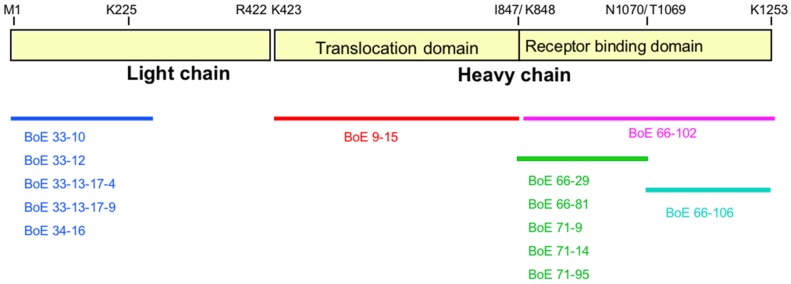
Epitope identification of anti-BoNT/E3 mAbs. Epitope location is based on antibody binding to recombinant toxin-GST fusion peptides. Diagram is drawn to approximate scale. The predicted transmembrane (K423 to I847) and receptor binding (K848 to K1253) domains are indicated.

A single mAb, BoE 9-15, was identified using the capture-capture screening method following fusions using the recHCP1-GST (the translocation domain) as the immunogen. Results of the peptide binding experiments clearly showed binding only to full-length recHC, recHCP1, and recHCP4 peptides suggesting a binding epitope localized to the translocation domain of BoNT, between K423-K848 (Figure S3E; Table 2). In contrast, six mAbs were identified using the capture-capture screen following immunization with the recHCP5-GST peptide (receptor binding domain). These mAbs segregated into two epitope groups, BoE 66-29, BoE 66-81, BoE 71-9, BoE 71-14, and BoE 71-95, bound recombinant peptides HCP2, HCP4, and HCP5 in addition to the recHC peptide. MAb BoE 66-106 bound the full length HC peptide as well as recombinant peptides HCP3 and HCP5. These data suggest that the former mAbs bound to the N-terminal half of the receptor binding domain and that mAb BoE 66-106 binds to the C-terminal half of the receptor binding domain (Figure S3H; Table 2). All of the mAbs epitope binding domain activity is summarized in Figure 3. 

### 2.4. Capture ELISA

**Figure 4:**
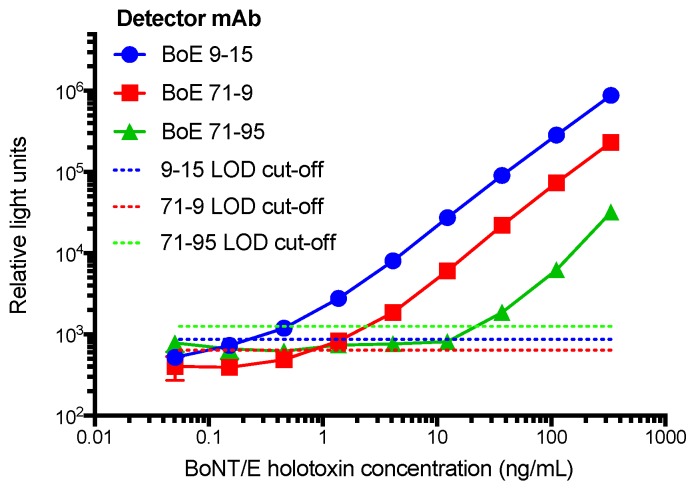
A capture ELISA for the detection of BoNT/E3 holotoxin using mAb BoE 66-29 as the capture antibody paired with biotinylated mAbs BoE 9-15, BoE 71-9, or BoE 71-95 as the detector antibody. Points represent average (*n* = 3) measurements, error bars are standard deviation, and the horizontal dashed lines equal the average of the zero spike plus three standard deviation (SD). Where not visible, error bars are within the symbols.

The objective of producing a large panel of mAbs is to then screen these for antibody pairs useful for generation of a sensitive capture ELISA to detect toxin. Therefore, each antibody presented in Table 1 was conjugated with biotin. Next, non-biotin labeled antibodies were individually absorbed onto 96-well microtiter plates, probed with varying amounts of neurotoxin holotoxin (BoNT/E3) and bound toxin measured using each of the biotin labeled mAbs. The three most sensitive assays coincidentally all used mAb BoE 66-29 as the capture antibody and were paired with either BoE 9-15, BoE 71-9, or BoE 71-95 as the detector antibody (Figure 4). These data suggest that the lower limit of detection (LOD) based on adding three standard deviations about the average signal of a zero toxin blank varied from 0.2 ng/mL for BoE 9-15, 0.9 ng/mL for BoE 71-9, and 20 ng/mL for BoE 71-95. 

### 2.5. Matrix Study

**Figure 5:**
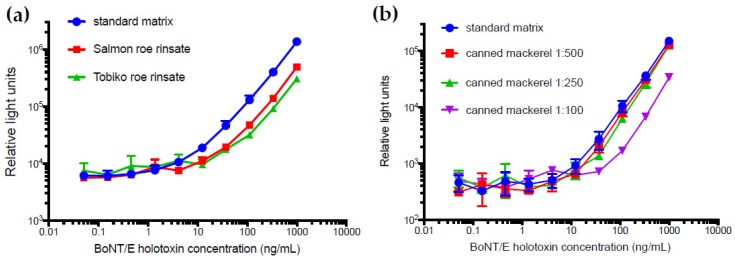
Analysis of matrix effects in the BoNT/E3 holotxin sandwich ELISA. The matrices (**a**) Salmon and Tobiko roe rinsates and (**b**) canned mackerel at dilutions of 1:100, 1:250, and 1:500 were spiked with BoNT/E3 holotoxin and are shown alongside the standard curve (blue circles) using 3% non-fat dried milk in tris buffered saline with Tween 20 (TBST) as the matrix. Where not visible, error bars are within the symbols.

To evaluate the utility of this capture ELISA for detecting toxin in food matrices, two types of roe and one canned fish product were tested. Since the toxin would likely be found on the surface of the roe, the specimens were not macerated, but instead rinsed with standard matrix buffer (3% non-fat dry milk in tris buffered saline with Tween 20 (TBST) to generate relevant food matrices. The food matrices did reduce the detectability, which resulted in an LOD of 10 ng/mL for the roe rinsates and for the 250-fold or greater dilutions of the brine from canned mackerel (Figure 5). While performing these matrix studies, the signal from the standard matrix curves generated each day continually dropped (Figure S4). This highlights the importance of running a standard curve each day, but also the potential instability of BoNT/E3 holotoxin, which can be detected by immunoassay.

## 3. Discussion

The more often performed method for generating mouse mAbs specific to a toxin, and in particular to BoNT/E, is to use the toxoid as the immunogen [15,16,17,18]. In theory, this method allows the antibodies to recognize any antigenic epitope, but in practice, the selected antibodies may not bind a diverse number of epitopes. As an alternative, here we demonstrated a strategy of utilizing peptide fragments as immunogens, which then limits each antibody to bind an antigenic epitope on that particular peptide. While both methods have been used successfully, they each have their limitations, such that epitopes that are now presented on the immunogen may not be present in the whole native toxin.

In light of immunogen presentation, other means to influence antigen selectivity is by the screening procedure used. Screening measures that are as similar as possible to the final application should be employed to identify the antibodies desired depending on the downstream application of these reagents. For this reason, in this work we utilized both direct binding assays (used to screen for anti-LC mAbs) and capture-capture binding assays (used to screen for anti-HC mAbs). Previous work yielding a mAb-mAb sandwich ELISA for BoNT/E utilized a direct binding screening method for selecting potential hybridomas [15]. In our work, it is interesting to note that the cross-reactivity of the mAbs generated to LC fragments is considerably more variable (Table 1). This may be due to the screening procedure or just an inherent result of the peptides used, but cannot be differentiated by these experiments. Nonetheless, we showed the production of new mAbs that can provide new insights when used individually or together depending on the desired applications. 

The most common applications of these mAbs are to neutralize toxin, identify structure–function relationships of the toxins, and to develop toxin detection assays. Our goal was to primarily develop a sandwich ELISA for detecting BoNT/E in suspected samples. Since mAbs are immunochemically homogeneous and more reproducible than pAbs, we sought to develop this sandwich ELISA using a pair of mAbs. In this work, we developed a mAb-mAb sandwich ELISAs for BoNT/E3 (Figure 4). Other applications of mAb-mAb sandwich-type assays have yielded improved LODs using other formats, such as microarrays [31]. In assays utilizing pAb sources [14,32,33], improved LODs were obtained and therefore it might be of interest for future research to combine multiple mAbs from this study to improve performance. Furthermore, using these mAbs in other formats and with different reporting molecules may also yield improved LODs.

While testing these mAbs for neutralization and other functionalities was outside the scope of this work, all of the mAbs described in this paper may be suitable for other applications. For example, most mAbs generated to date recognize the HC of BoNT/E [15]. Here we describe five new mAbs that bind the LC—as determined by Western blot or binding of the recLC fragment by ELISA (Figure 2 and Table 2). Further characterization of all of these mAbs could include determining their ability to detect BoNT/E complex, to detect BoNT/E subtypes E1–12, and if they are capable of neutralizing toxin.

In instances when immunoassays are sought to be used with food matrices, matrix effects may represent a major hurdle to the feasibility of the assay. Due to the documented prevalence of BoNT/E in fish products, we sought to evaluate our sandwich ELISA with roe and canned fish products. We observed a 50-fold decrease in LOD from 0.2 ng/mL in buffer to 10 ng/mL in fish product matrices. This study aligns with previous studies by demonstrating that there is commonly a decrease in the amount of toxin detected when toxin is extracted from a food matrix [15,31,33]. For instance, in a study examining 30 food matrices (none of which were fish products) they report a 12-fold decrease in LOD from 163 pg/mL in buffer to 2 ng/mL in food matrices [33]. An evaluation of ELISA kits demonstrated a LOD in smoked Salmon matrix of 60 pg/mL [34]. While decreased sensitivity impacts the utility of immunoassays as a quantitative method for measuring toxin, as a screening tool, if any toxin is present it would generate a positive signal and suggest further analysis. Although the matrix might impede detection, our study substantiates that matrix interferences in ELISA are manageable with a simple dilution step [24] (Figure 5b). It was speculated that a lethal oral amount of botulinum toxin for a 70-kg human would be 70 μg [35]. While the sensitivity of this assay is not likely suitable for human biospecimen diagnostic purposes, it is approaching relevant levels for food analysis. To improve sensitivity, the mAbs could be used in other purification/concentration steps (such as beads coated with anti-BoNT/E mAbs) prior to food testing.

In summary, this work demonstrates the production of new mAbs to BoNT/E3 and when paired together generated a sensitive sandwich ELISA for the detection of BoNT/E3. This strategy utilized peptide fragments as immunogens to induce antibodies selective for various fragments of BoNT/E3. Additional peptides not used as immunogens were used to further narrow down the binding regions of the induced antibodies. Furthermore, a capture-capture screening procedure was used to isolate antibodies that functionally bound toxin in solution, as opposed to binding to an immobilized target, which may distort particular epitopes. Ultimately, this work yielded a mAb-mAb sandwich ELISA that can detect BoNT/E3 holotoxin at 0.2 ng/mL in buffer and 10 ng/mL in spiked fish products.

## 4. Materials and Methods

### 4.1. Reagents

Solutions at 1 mg mL^−1^ of BoNT serotypes A–G, the 150 kDa serotype E holotoxin (BoNT/E) produced by *Clostridium botulinum* Strain E3/Alaska, and anti-BoNT/E rabbit pAbs were purchased from Metabiologics Inc. (Madison, WI, USA). Nicked BoNT/E3 was obtained from List Biological Laboratories (Campbell, CA, USA). Total genomic DNA from *C. botulinum* (Strain E3/Alaska) was generously provided by Eric Johnson (University of Wisconsin, Madison, WI, USA). All toxins were handled in a BSL-2 biosafety cabinet and all materials and waste solutions were treated before disposal. 

Bovine serum albumin (BSA), ovalbumin (OVA), goat anti-mouse immunoglobulin G conjugated to horseradish peroxidase (IgG-HRP), polyoxyethylene sorbitan monolaurate (Tween-20), Sigma Adjuvant System, Protein-G conjugated Sepharose, and the following buffers: 0.01 M phosphate buffered saline (PBS), 0.138 M NaCl, 0.0027 M KCl, pH 7.4, and 0.02 M TRIS-buffered saline (TBS), 0.9% NaCl, pH 7.4 were purchased from Sigma Chemical Co. (St. Louis, MO, USA). Goat anti-mouse IgG Fc gamma was purchased from Millipore (Billerica MA). Black and clear Maxisorp 96-well Nunc microtiter plates were obtained from PGC Scientific (Gaithersburg, MD, USA). BCA kit, EZ-Link Sulfo-NHS-LC-Biotin kit, SuperSignal Femto Max Sensitivity substrate, and SuperSignal West Dura Extended Duration substrate were purchased from Pierce Inc. (Rockford, IL, USA). SBA Clonotyping System-HRP was purchased from Southern Biotech (Birmingham, AL, USA). Enhanced K-Blue TMB substrate and Red Stop Solution were obtained from Neogen Corporation (Lexington, KY, USA). Non-fat dry milk (NFDM), fresh Tobiko roe, fresh Salmon roe, and canned in brine mackerel were purchased from a local food store.

Commercial enzymes (Phusion High-Fidelity DNA Polymerase, BamHI, XhoI, T4 polynucleotide kinase (3’ phosphatase minus) and T4 DNA ligase were purchased from New England BioLabs, Inc. (Bethesda, MD, USA). Plasmid construction and manipulation were performed using pCR4-TOPO vector and *Escherichia coli* TOP10 cells from Invitrogen (Carlsbad, CA, USA) Plasmid pGS-21 and QuickClean 5M kits (Miniprep, PCR purification, and Gel extraction) were from GenScript Corp. (Piscataway, NJ, USA). All automated DNA sequencing was performed on a 3730 DNA Analyzer using the BigDye Terminator Version 3.1 cycle sequencing kit and XTerminator reagents purchased from Applied Biosystems, (Foster City, CA, USA). Primers designed specifically for this project were purchased from Integrated DNA Technologies (Coralville, IA, USA) and are shown in Table 3.

### 4.2. Recombinant BoNT/E3-GST Fusion Proteins

The expression and purification of all GST-fusion proteins was performed as previously described [30] with the following modifications. Total genomic DNA from *C. botulinum* Strain E3/Alaska was used as a template to amplify the fragments of the light and heavy chains (LC, LCP1, LCP2, HC, HCP1, HCP2, HCP3, HCP4, HCP5) using the primers indicated (see Figure 1 and Table 1). Stop codons (TAA) were introduced when not present within the genomic DNA of the cloned region. The resulting BoNT/E3 DNA fragments were cloned into plasmid pCR4-TOPO and sequenced using M13F and M13R primers. The pCR4-derived plasmids were digested using restriction enzymes BamHI and XhoI, purified, and then ligated into BamHI- and XhoI-digested pGS-21a to yield the correspondingly named pGS plasmid (e.g., pGS-HCP1 for fragment HCP1). All pGS-21a-derived plasmids were sequenced using pGS-F and pGS-R primers, to confirm the correct integration of the BoNT/E3 fragment into the vector. The recombinant DNA methods used in this study were approved by the Institutional Biosafety Committee.

### 4.3. Monoclonal Antibody Procedure

Monoclonal antibody production was as previously described [29,36]. Briefly, solutions of three peptide fragments, separately, at the concentrations indicated (LC, 94 µg/mL; HCP1, 79 µg/mL; HCP5, 176 µg/mL) were mixed with Sigma Adjuvant System according to the manufacturer’s instructions. Three groups of five female BALB/cJ mice (Simonsen Laboratories, Gilroy, CA, USA) were immunized three times at two-week intervals by intraperitoneal injection (i.p.) with 100 µL of each antigen-adjuvant solution. Two weeks after the third injection, serum was obtained from each mouse and evaluated for anti-BoNT/E3 antibodies via direct binding ELISA screens. Mice were injected i.p. with 2 µg of the appropriate peptide fragment in PBS three days prior to being euthanized and cell fusion. Supernatants from cell fusion plates were subjected to screening either by direct binding ELISA (for mice immunized with the recBoNT/E3-LC-GST peptide fragments) or by a capture-capture ELISA screen (for the mice immunized with the recBoNT/E3-HCP1-GST and recBoNT/E3-HCP5-GST peptide fragments).

The Institutional Animal Care and Use Committee of the United States Department of Agriculture, Western Regional Research Center approved the experimental procedures used in these studies (protocol #’s: 04-1-H-05; 09-3). All animal experiments and husbandry involved in the studies presented in this manuscript were conducted under the guidelines of the U.S. Government Principles for the Utilization and Care of Vertebrate Animals Used in Testing, Research and Training.

### 4.4. Screening Methods

#### 4.4.1. Directing Binding ELISA

Direct binding ELISA screens of the recBoNT/E3-LC-GST immunized mice were performed as previously described [36], using black microtiter plates coated with 50 µL per well of a 0.1 µg/mL solution of BoNT/E3 in 0.05 M sodium carbonate buffer, pH 9.6, overnight at 4°C. The solution was aspirated and then blocked by adding 300 µL per well of 3% NFDM in TBS containing 0.05% Tween-20 (NFDM-TBST) and incubated for 1 h at 37 °C. The plates were washed once with TBST, and then the cell culture supernatants were added. After another incubation at 37 °C for 1 h, the plates were washed 3× with TBST. Then 50 µL of a 1 µg/mL solution of goat anti-mouse HRP-conjugated pAb was added and the plates incubated at 37 °C for 1 h. After washing 3× with TBST, the plates were visualized using SuperSignal West Dura Extended Duration Substrate according to the manufacturer’s instructions. The plates were incubated for 3 min at room temperature and luminescent counts recorded using a Victor^3^ Multilabel Counter (PerkinElmer Inc., Waltham, MA, USA).

#### 4.4.2. Capture-Capture ELISA

Fusions of the recBoNT/E3-HCP1-GST and recBoNT/E3-HCP5-GST immunized mice were screened using a capture-capture ELISA screen as previously described with minor modifications [37,38]. Briefly, 50 µL per well of all solutions were used. Black microtiter plates were coated with a 1 µg/mL solution of goat anti-mouse IgG Fc gamma in 0.05 M sodium carbonate buffer, pH 9.6 overnight at 4 °C. The IgG solution was aspirated and non-coated sites blocked by adding 300 µL per well of 3% NFDM-TBST and the plates were incubated for 1 h at 37 °C. The plates were washed once with TBST, then cell culture supernatants were added and the plates were incubated at 37 °C for 1 h. The plates were washed three times with TBST, then a solution of BoNT/E3 in NFDM-TBST (50 ng/mL) was added and the plates were incubated at 37 °C for 1 h. Plates were washed three times as before, then a 1 µg/mL solution of anti-BoNT/E rabbit pAbs in NFDM-TBST was added and the plates were incubated at 37 °C for 1 h. Plates were washed three times as before, then a 1 µg/mL solution of goat anti-rabbit HRP-conjugated pAbs was added and the plates were incubated at 37 °C for 1 h. Plates were again washed three times, and binding was visualized as described above.

Cells from wells giving positive signals for antibody production by either screening method were cloned by limiting dilution. Cells were then expanded and small amounts (usually less than 10 mL) of ascites fluids obtained (Covance Research Products, Inc., Denver, PA, USA) or 1–200 mL of spent hybridoma media was produced. MAbs were purified by affinity chromatography on Protein-G (for IgG) Sepharose. Bound mAbs were eluted with 0.1 M glycine-HCl, pH 2.7 and dialyzed overnight against PBS. Protein concentrations were determined using the microplate BCA method suggested by the manufacturer. Then, the mAbs were conjugated to biotin using EZ-Link Sulfo-NHS-LC-Biotin as described by the manufacturer using a 50-fold molar excess of the biotin reagent. MAbs were stored at 4 °C until needed.

### 4.5. Antibody Isotyping, Western Blotting, Peptide Binding

The isotype of each mAb was determined using the SBA Clonotyping System-HRP in ELISA format, according to manufacturer’s instructions. Western blotting was performed as previously described [36] using nicked BoNT/E3 using both reduced and non-reduced conditions. Binding of each antibody to the recBoNT/E3-GST peptides was examined by direct binding ELISA. The wells of clear microtiter plates were coated with each GST-fusion protein (5 µg/mL in 0.05 M sodium carbonate buffer, pH 9.6), incubated overnight at 4 °C, blocked with 3% NFDM-TBST. Purified mAb was added to each well and incubated for 1 h at 37 °C. The plates were washed 3× with TBST, then 50 µL of a 1 µg/mL solution of goat anti-mouse HRP-conjugated pAb was added and the plates incubated at 37 °C for 1 h. Plates were again washed three times, then K-Blue TMB substrate was added (100 µL per well) and incubated with agitation for 5 min at room temperature. Stop solution was added (100 µL per well), and absorbance at 450 nm was measured using a VersaMax microplate reader (Molecular Devices, Sunnyvale, CA, USA). Each antibody was tested in triplicate. In some experiments SuperSignal West Dura Extended Duration Substrate was substituted for K-Blue and plates read on the Victor^3^ as described above.

### 4.6. Binding of Antibodies to BoNT Serotypes A Through G

Black microtiter plates were coated with the different serotypes of BoNT, A through G for direct binding ELISA analysis as described above. Purified anti-BoNT/E3 mAb at a concentration of 10 µg/mL in NFDM-TBST was added to each BoNT serotype, and incubated for 1 h at 37 °C. The plates were washed, then a 1 µg/mL solution of goat anti-mouse HRP-conjugated pAb was added and the plates were incubated at 37 °C for 1 h. Plates were again washed three times, and binding was visualized using SuperSignal West Dura Extended Duration Substrate as described above. Each antibody was assayed against each serotype in triplicate.

### 4.7. Sandwich Assay Procedure

When performing the sandwich assay, for every mAb, the unlabeled mAb was used as the capture antibody and the biotinylated form was used as the detector antibody. Each mAb was tested in combination with all of the mAbs to identify pairs that can detect the BoNT/E3 toxin in the solution phase. Black plates were coated with 100 µL of mAb at 2 µg/mL in 0.05 M sodium carbonate buffer, pH 9.6, incubated overnight at 4 °C, and then blocked with 3% NFDM-TBST. The blocking solution was removed and BoNT/E3 toxin in 3% NFDM-TBST was added at an initial concentration of 500 ng/mL and serially diluted two-fold, including wells with no toxin. Next, biotinylated-mAb was added in triplicate at 1 µg/mL, 100 µL/well in 3% NFDM-TBST. Plates were incubated for 1 h at 37 °C, washed 6× with TBST, and visualized using SuperSignal West Dura Extended Duration Substrate as described above. 

When the most sensitive combination of capture mAb and detector mAb-biotin were selected, the final sandwich assays were performed in triplicate using BoNT/E3 holotoxin at a starting concentration of 1000 ng/mL, serially diluted three-fold, including wells with no toxin, and visualized using SuperSignal Femto Max Sensitivity substrate. Limit of detection cut-off values were determined using three times the standard deviation of the wells with no toxin. 

### 4.8. Preparation of Spiked Food Matrices

Since fish and fishery products are frequently cited as sources for serotype E related foodborne botulism cases, we tested one canned fish product (mackerel in brine) and two types of fish roe (Tobiko and Salmon) to determine to what extent the matrices would interfere with toxin detection in our newly developed sandwich ELISA. For the canned mackerel, the liquid fraction was poured into a conical tube and centrifuged for 20 minutes at 4000× g. The oil and fat layers were discarded, while the aqueous fraction was collected and used as the sample matrix. For each roe specimen, separately, 4 g of roe was weighed and rinsed with 40 mL of 3% NFDM-TBST. This rinsate was collected and used as the sample matrix. As described above, the sandwich assay procedure with the most sensitive combination of mAbs was used (BoE 66-29 as capture and BoE 9-15 as detector), but this time the BoNT/E3 holotoxin was diluted in sample matrix to generate standard curves. The assays were performed in triplicate using BoNT/E3 at a starting concentration of 1000 ng/mL, serially diluted three-fold into a sample matrix, including wells with no toxin. The sample matrices were evaluated as is (i.e., neat) for the roe rinsates, and at dilutions of 1:100, 1:250, and 1:500 with 3% NFDM-TBST for the aqueous brine matrix. Experiments were visualized using SuperSignal Femto Max Sensitivity substrate and data analyzed using GraphPad Prism 7 Software (La Jolla, CA, USA). 

## Figures and Tables

**Table 1:** Characterization of monoclonal antibodies (mAbs) developed against BoNT/E3.

Antibody	Immunogen ^1^	Myeloma Fusion Partner	Fusion Screen	Isotype ^2^	Peptides Bound ^1^	Binding Domain	Serotype Specificity ^3^
BoE 33-10	recBoNT/E-LC	Sp2/0	Direct binding	IgG2b	LC, LCP1	M1–K225	E, F > B > C, D, G
BoE 33-12	recBoNT/E-LC	Sp2/0	Direct binding	IgG1	LC, LCP1	M1–K225	E, F > B > C, D, G, A
BoE 33-13-17-9	recBoNT/E-LC	Sp2/0	Direct binding	IgG1	LC, LCP1	M1–K225	E, F > B > C, D, G
BoE 33-13-17-4	recBoNT/E-LC	Sp2/0	Direct binding	IgG1	LC, LCP1	M1–K225	E >> B
BoE 34-16	recBoNT/E-LC	Sp2/0	Direct binding	IgG2a	LC, LCP1	M1–K225	E, F > B > C, D, G
BoE 9-15	recBoNT/E-HCP1	Sp2/0	Capture/Capture	IgG2b	HC, HCP1, HCP4	K423– I847	E
BoE 66-29	recBoNT/E-HCP5	P3XU.1	Capture/Capture	IgG1	HC, HCP2, HCP4, HCP5	K848–T1069	E
BoE 66-81	recBoNT/E-HCP5	P3XU.1	Capture/Capture	IgG2b	HC, HCP2, HCP4, HCP5	K848–T1069	E
BoE 66-106	recBoNT/E-HCP5	P3XU.1	Capture/Capture	IgG1	HC, HCP3, HCP5	N1070– K1253	E >> B
BoE 71-9	recBoNT/E-HCP5	Sp2/0	Capture/Capture	IgG1	HC, HCP2, HCP4, HCP5	K848–T1069	E (weak)
BoE 71-14	recBoNT/E-HCP5	Sp2/0	Capture/Capture	IgG1	HC, HCP2, HCP4, HCP5	K848–T1069	E
BoE 71-95	recBoNT/E-HCP5	Sp2/0	Capture/Capture	IgG1	HC, HCP2, HCP4, HCP5	K848–T1069	E

^1^ Refer to Figure 1 for definition of peptide designations. ^2^ All with kappa light chains. ^3^ Binding specificity determined using immobilized toxin by ELISA (see Figures S1 and S2).

**Table 2:** Binding signals for anti-BoNT/E3 mAbs binding to recombinant peptides corresponding to those shown in Figure 1. Peptides were absorbed onto microtiter wells and probed with specific mAbs. Binding was determined by measuring absorbency (OD, optical density) as described in the methods.

mAb	recLC	recLCP1	recLCP2	recHC	HCP1	HCP2	HCP3	HCP4	HCP5
BoE 33-10	1.71 ± 0.04	1.79 ± 0.03	0.07 ± 0.00	0.07 ± 0.01	0.07 ± 0.01	0.08 ± 0.01	0.07 ± 0.0	0.08 ± 0.01	0.07 ± 0.00
BoE 33-12	2.06 ± 0.01	2.05 ± 0.03	0.06 + 0.00	0.06 + 0.00	0.06 ± 0.01	0.08 ± 0.02	0.05 ± 0.00	0.08 ± 0.04	0.05 ± 0.03
BoE 33-13-17-4	1.05 ± 0.09	1.25 ± 0.08	0.13 ± 0.01	0.09 ± 0.01	0.08 ± 0.01	0.13 ± 0.02	0.16 ± 0.00	0.1 ± 0.01	0.13 ± 0.02
BoE 34-16	2.44 ± 0.04	2.46 ± 0.03	0.88 ± 0.02	0.08 ± 0.01	0.07 + 0.01	0.09 ± 0.01	0.07 ± 0.00	0.08 ± 0.01	0.06 + 0.01
BoE 9-15	0.11 ± 0.08	0.07 ± 0.01	0.06 ± 0.02	1.17 ± 0.02	1.37 ± 0.01	0.07 ± 0.17	0.06 ± 0.02	1.2 ± 0.01	0.05 + 0.01
BoE 66-29	0.05 ± 0.00	0.05 ± 0.00	0.05 ± 0.01	0.59 ± 0.02	0.05 ± 0.01	0.78 ± 0.02	0.05 + 0.15	0.7 ± 0.02	0.70 ± 0.02
BoE 66-81	0.06 ± 0.03	0.05 ± 0.00	0.05 ± 0.02	0.55 ± 0.00	0.05 ± 0.12	0.91 + 0.04	0.06 ± 0.03	0.78 ± 0.08	0.7 ± 0.003
BoE 66-106	0.06 ± 0.01	0.06 ± 0.01	0.06 + 0.01	0.62 ± 0.02	0.06 ± 0.01	0.06 ± 0.01	0.78 ± 0.02	0.07 ± 0.01	0.76 ± 0.03
BoE 71-9	0.06 ± 0.01	0.06 ± 0.01	0.06 ± 0.00	0.23 ± 0.01	0.05 ± 0.01	0.77 ± 0.06	0.06 ± 0.01	0.61 + 0.03	0.51 ± 0.02
BoE 71-14	0.06 ± 0.01	0.01 ± 0.01	0.06 ± 0.01	0.55 ± 0.04	0.06 ± 0.01	0.89 ± 0.04	0.10 ± 0.01	0.78 ± 0.03	0.74 + 0.03
BoE 71-95	0.06 ± 0.01	0.06 ± 0.00	0.05 + 0.00	0.32 ± 0.01	0.06 ± 0.01	0.87 ± 0.07	0.06 ± 0.01	0.50 + 0.03	0.83 ± 0.05

Signal values are average and standard deviation for three replicate wells; bold text indicates significant binding events with values >0.2 OD. Background binding signals for non-coated and GST-coated wells never exceeded 0.13 ± 0.02. BoE 33-13-17-9 not shown.

**Table 3:** Primers designed for this study. Sites for restriction enzymes BamHI (GGATCC) and XhoI (CTCGAG) are shown underlined. Stop codons are shown in bold, in the 3’ to 5’ (TTA) orientation.

Primer Name	Sequence
AlaskaLCF	TAGC GGA TCC ATG CCA AAA ATT AAT AGT TTT AAT TAT AAT
AlaskaLCF2	TAGC GGA TCC GGG ATT ACT ACA ACT TGT ATT ATA
AlaskaLCR	TAGC CTC GAG TTA CCT TAT GCC TTT TAC AGA AAC AA
AlaskaLCR2	TAGC CTC GAG TTA TTT AGC CCC ATA TAG TCC ATG T
AlaskaHCF	TAGC GGA TCC AAA TCA ATA TGT ATC GAA ATA AAT AAT
AlaskaHCF2	TAGC GGA TCC AAA AGT AGT TCA GTT TTA AAT ATG AG
AlaskaHCF3	TAGC GGA TCC AAT ATT TTG AAG GAT TTT TGG GGA
AlaskaHCR	TAGC CTC GAG TTA TTT TTC TTG CCA TCC ATG TT
AlaskaHCR1	TAGC CTC GAG TTA AAT TCT CTT AAA GAA TTT ATT AAA ATA T
AlaskaHCR2	TAGC CTC GAG TTA TGT ATT AGG TTC ATT GCT ATA TAA A

## Supplementary Materials

The following are available online at https://www.mdpi.com/2072-6651/11/7/407/s1, Figure S1: Binding of LC-Specific anti-BoNT/E3 mAbs (10 µg/mL) to toxin serotypes A–G, used as coating antigens in a direct binding ELISA, Figure S2: Binding of HC-specific anti-BoNT/E3 mAbs (10 µg/mL) to toxin serotypes A–G, used as coating antigens in a direct binding ELISA, Figure S3: Direct binding ELISA of anti-BoNT/E3 mAbs to recombinant peptide fragments. Different antibodies are shown in A–K, Figure S4: Chemiluminescent signal intensity of the ELISA over time at the maximal concentration (1000 ng/mL of BoNT/E3 holotoxin) tested on the standard curve.
