# Supplementary material for: Development and Characterization of Monoclonal Antibodies to Botulinum Neurotoxin Type E
Source: Toxins (Basel). 2019 Jul 13;11(7):407. doi: 10.3390/toxins11070407 (PMC6669634; doi:10.3390/toxins11070407)
Supplement: Supplementary file 1 [file toxins-11-00407-s001.pdf]

Supplemental Materials: Development and Characterization of Monoclonal Antibodies to Botulinum Neurotoxin Type E

Candace Bever, Miles Scotcher, Luisa W. Cheng Robert Hnasko and Larry H. Stanker

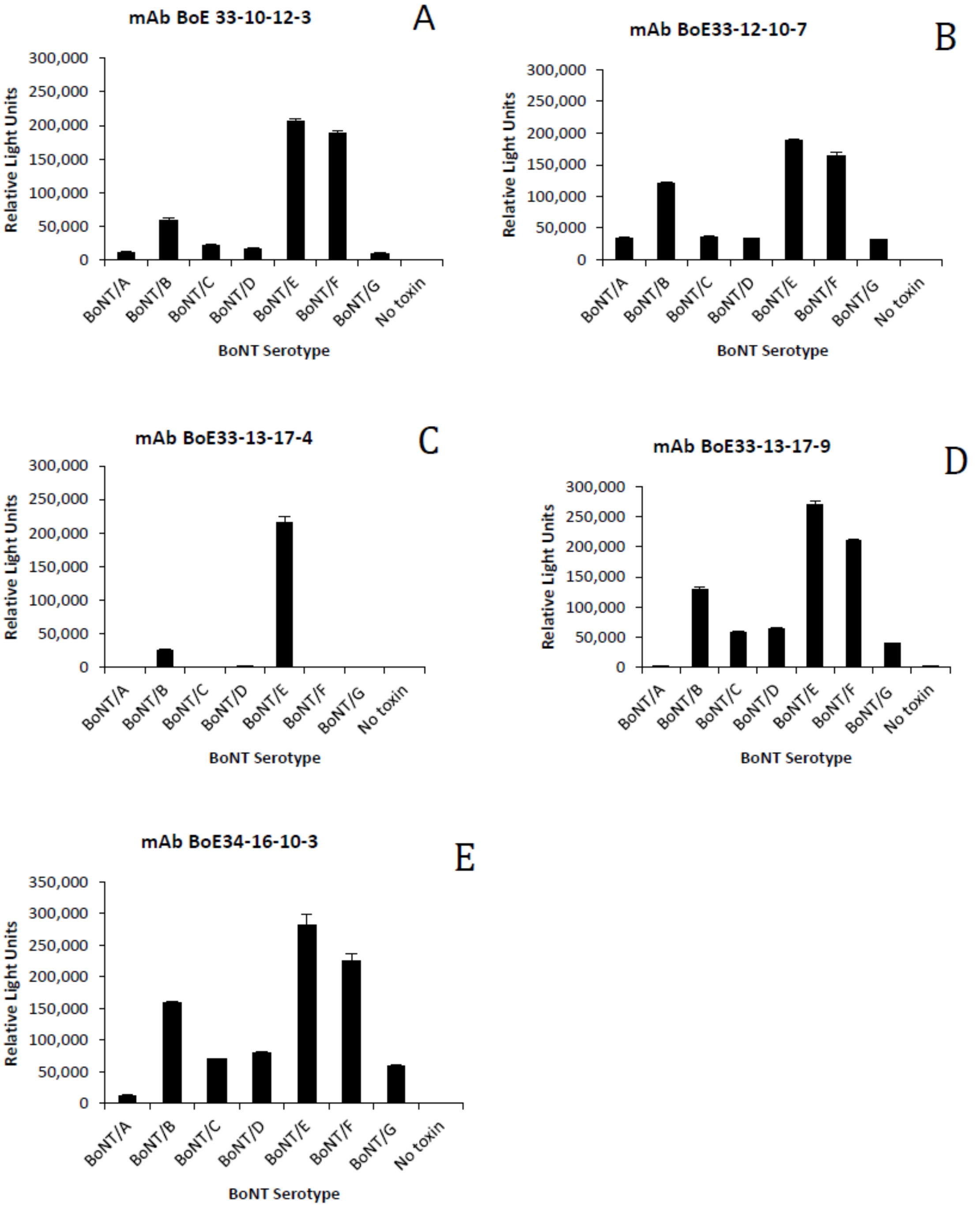

Figure S1. Binding of Lc-Specific anti-BoNT/E mAbs (10 µg/mL) to Toxin Serotypes A–G, used as coating antigens in a direct binding ELISA. Different antibodies are shown in A–E. Each bar is an average of triplicate analysis. Binding activity was determined by chemiluminescence intensity.

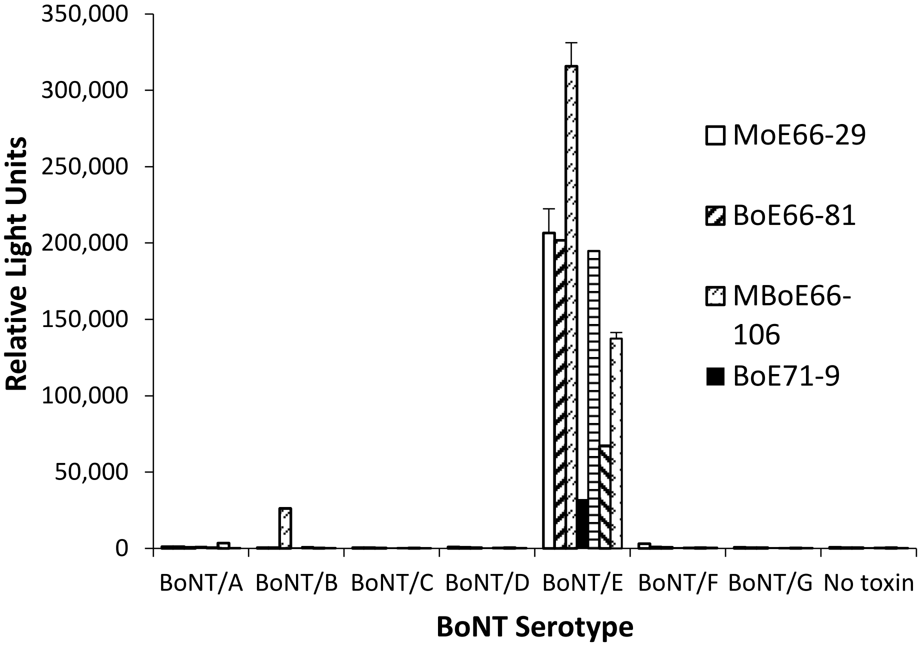

Figure S2: Binding of Hc-Specific anti-BoNT/E mAbs (10 µg/mL) to Toxin Serotypes A-G, used as coating antigens in a direct binding ELISA. Each bar is an average of triplicate analysis. Binding activity was determined by chemiluminescence intensity.

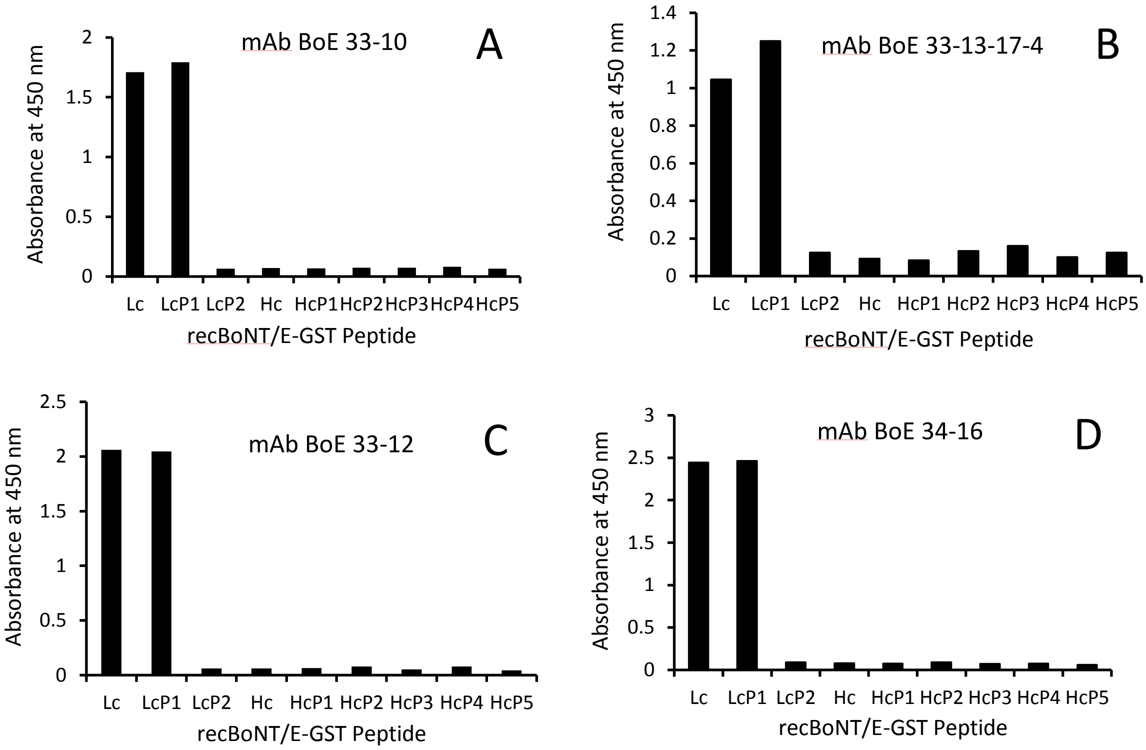

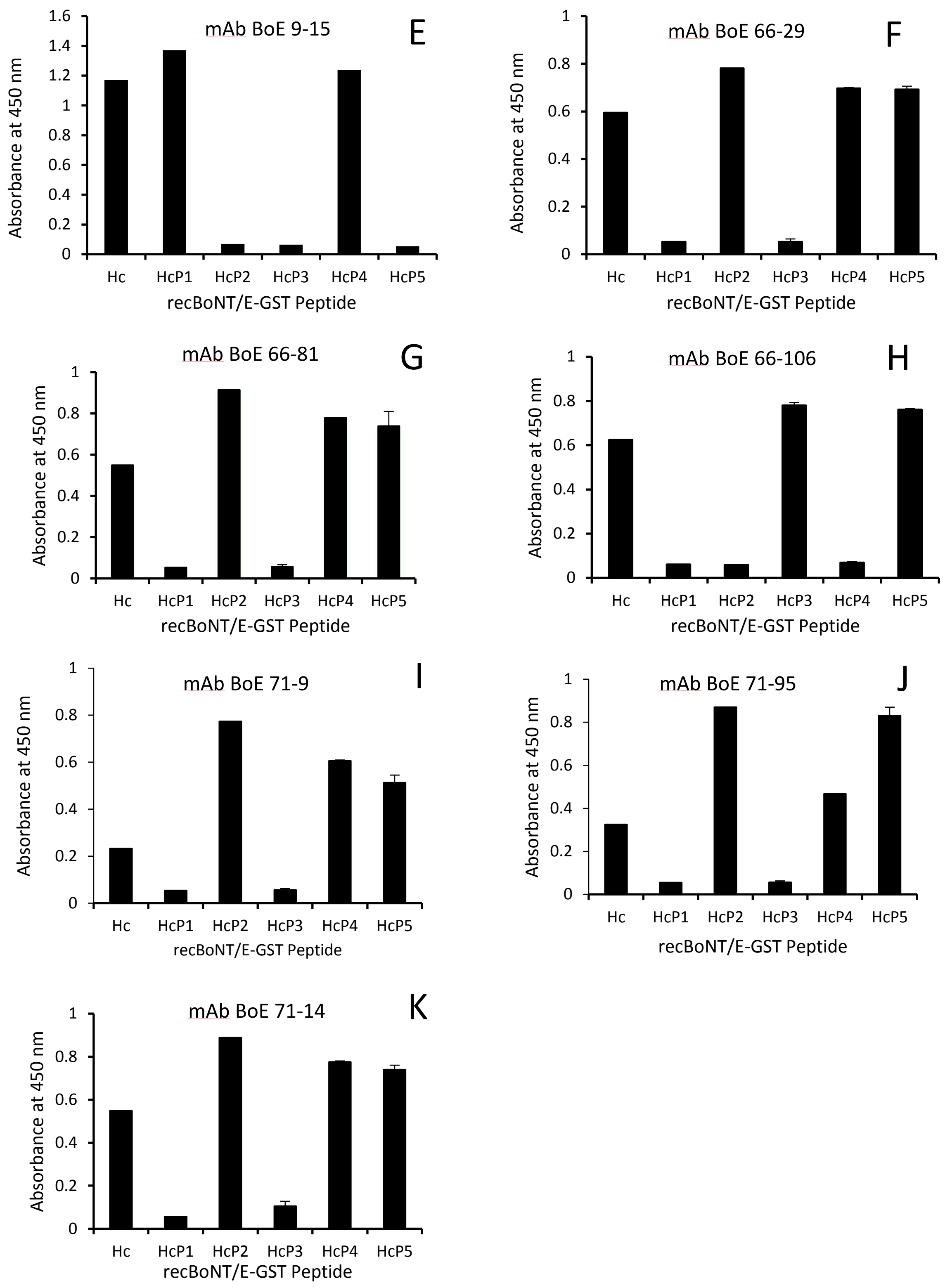

Figure S3: Direct binding ELISA of anti-BoNT/E mAbs to recombinant peptide fragments. Different antibodies are shown in A-K. Binding activity was determined as colorimetric absorbance.

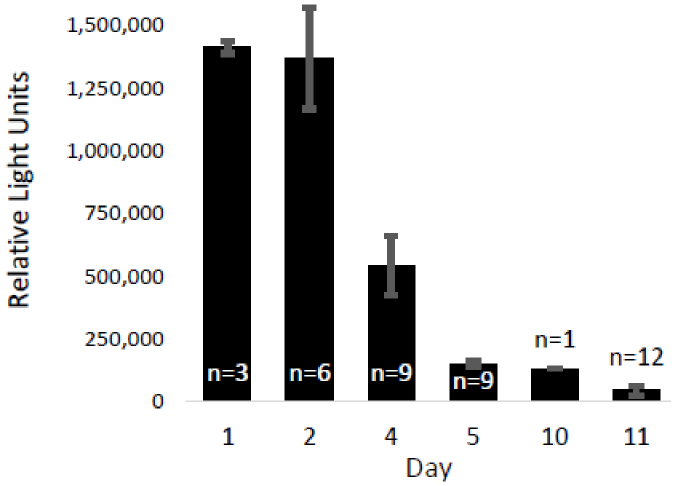

Figure S4. Chemiluminescent signal intensity of the ELISA over time at the maximal concentration (1,000 ng/mL of BoNT/E holotoxin) tested on the standard curve. The number of replicates is indicated for each bar.
